# Global Metabolomic Characterizations of *Microcystis* spp. Highlights Clonal Diversity in Natural Bloom-Forming Populations and Expands Metabolite Structural Diversity

**DOI:** 10.3389/fmicb.2019.00791

**Published:** 2019-04-16

**Authors:** Séverine Le Manach, Charlotte Duval, Arul Marie, Chakib Djediat, Arnaud Catherine, Marc Edery, Cécile Bernard, Benjamin Marie

**Affiliations:** UMR 7245 MNHN/CNRS Molécules de Communication et Adaptation des Micro-organismes, Muséum National d’Histoire Naturelle, Paris, France

**Keywords:** cyanobacteria blooms, secondary metabolites, chemiodiversity, mass spectrometry, aquatic environment

## Abstract

Cyanobacteria are photosynthetic prokaryotes capable of synthesizing a large variety of secondary metabolites that exhibit significant bioactivity or toxicity. *Microcystis* constitutes one of the most common cyanobacterial genera, forming the intensive blooms that nowadays arise in freshwater ecosystems worldwide. Species in this genus can produce numerous cyanotoxins (i.e., toxic cyanobacterial metabolites), which can be harmful to human health and aquatic organisms. To better understand variations in cyanotoxin production between clones of *Microcystis* species, we investigated the diversity of 24 strains isolated from the same blooms or from different populations in various geographical areas. Strains were compared by genotyping with 16S-ITS fragment sequencing and metabolite chemotyping using LC ESI-qTOF mass spectrometry. While genotyping can help to discriminate among different species, the global metabolome analysis revealed clearly discriminating molecular profiles among strains. These profiles could be clustered primarily according to their global metabolite content, then according to their genotype, and finally according to their sampling location. A global molecular network of all metabolites produced by *Microcystis* species highlights the production of a wide set of chemically diverse metabolites, including a few microcystins, many aeruginosins, microginins, cyanopeptolins, and anabaenopeptins, together with a large set of unknown molecules. These components, which constitute the molecular biodiversity of *Microcystis* species, still need to be investigated in terms of their structure and potential bioactivites (e.g., toxicity).

## Introduction

During recent decades, the frequency and the intensity of cyanobacteria proliferation occurring in continental aquatic ecosystems have increased due to climate and anthropogenic changes (Carey et al., 2012; Sukenik et al., 2015; Paerl, 2018). The resulting massive cyanobacteria “blooms” threaten aquatic ecosystem function through various processes, including: (i) alteration of the trophic network, (ii) decrease in the light penetrating the water column, (iii) decrease in available dissolved oxygen, and (iv) production of various secondary metabolites that are potentially toxic for living organisms (Carmichael, 2008). Indeed, various cyanobacterial genera can synthetize a wide range of secondary metabolites (Welker et al., 2012; Shih et al., 2013), with noticeable bioactivity and high toxicity, causing potential harm to human and aquatic organism populations (Codd et al., 2005; Pearson et al., 2010). These metabolites are also believed to affect the proliferative capability of the cyanobacteria themselves (Guljamow et al., 2017).

**FIGURE 1:**
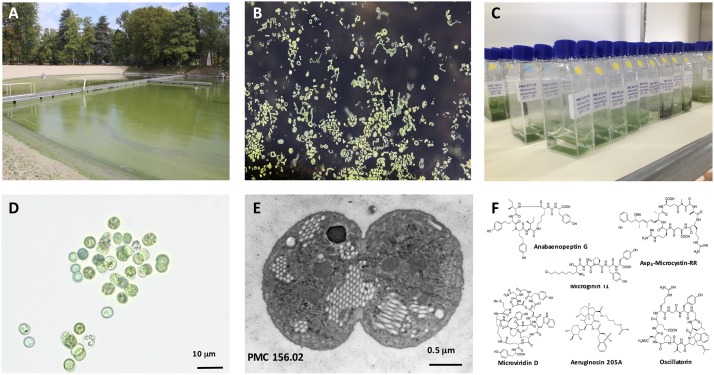
*Microcystis* spp. General view of a representative intense *Microcystis* sp. bloom in a recreational pound (Champs-sur-Marne, © B. Marie) **(A)**. Macrograph of *Microcystis* colonies at surface water (© B. Marie) **(B)**. Example of 15-mL vessels containing the monoclonal strains of *Microcystis* spp. maintained in the Paris’ Museum Collection (PMC) of cyanobacteria (MNHN, Paris, © C. Duval) **(C)**. Example of micrograph of the isolated monoclonal culture of the *Microcystis aeruginosa*, where scale bare represents 10 μm (© C. Duval) **(D)**. Representative picture of *Microcystis aeruginosa* cell from PMC 156.02 strain (here in division) under transmission electron microscope, where scale bare represents 0.5 μm (© C. Djediat) **(E)**. General structures of various cyanobacterial metabolites belonging to the microcystin, anabaenopeptin, microginin, microviridin, aeruginosin and oscillatorin families **(F)**.

*Microcystis* represents one of the most proliferative bloom-forming cyanobacterial genera (Bishop et al., 1959; Figure 1). It has been reported in more than 108 countries and on all continents (Šejnohová and Maršálek, 2012; Harke et al., 2016; Ma et al., 2016). Previous documentations had only reported *Microcystis* in less than 30 countries (Zurawell et al., 2005). This suggests that species within this genus are currently proliferating and largely dominating freshwater phytoplankton communities in temperate and tropical areas. In temperate ecosystems, *Microcystis* species can even overwinter in the benthos, rise from the epilimnion during the summer and can accumulate to form intensive blooms and even scums on the surface (Harke et al., 2016).

Some important features of *Microcystis* species specifically favor their worldwide expansion (Paerl et al., 2011). These features include the capability to regulate their buoyancy, their winter storage strategy at the bottom of the water column, their phosphate (P) and nitrogen (N) uptake capacities, and their resistance to zooplankton grazing. Indeed, *Microcystis* species exhibit competitive advantages during nutrient limitation or environmental warming compared to other cyanobacteria or microalgae. In addition, many *Microcystis* strains can produce a multitude of bioactive secondary metabolites, including the potent hepatotoxins microcystins (MCs). Then, the persistence of their proliferation poses local risks to those using contaminated water resources for consumption, recreational activities, agriculture, or fisheries (Codd et al., 2005). Beyond MCs, other potentially toxic compounds produced by *Microcystis* species have also been reported to be deleterious for aquatic organisms, as they potentially inhibit the grazing capability of herbivorous planktonic organisms (Harke et al., 2017).

So far, 11 secondary-metabolite biosynthetic gene clusters encoding non-ribosomal peptide synthase (NRPS) and/or polyketide synthase (PKS) and two other clusters encoding ribosomes were detected within ten *Microcystis* genomes (Humbert et al., 2013). Seven of these clusters encode enzymes involved in the biosynthesis of already known metabolites (such as microcystins, aeruginosins, cyanopeptolins, microginins, anabaenopeptins, cyanobactins, and microviridins), whereas the six remaining clusters seem to encode different enzymes responsible for the biosynthesis of yet-uncharacterized compounds. However, the relationship between cyanobacterial biomass and metabolite concentration in the environment appears neither systematic nor linear (Briand et al., 2012; Liu et al., 2016). Indeed, the production of metabolites, such as microcystins, by *Microcystis* blooms, depends not only on cyanobacterial biomass, but also on the dynamics of the ratio between potentially producing and non-producing genotypes (Via-Ordorika et al., 2004).

Despite recent advances in the description of the biosynthetic pathways involved in cyanobacterial metabolite synthesis (Wang et al., 2014; Dittmann et al., 2015), the biological functions and the ecological roles of these molecules are still not fully understood (Holland and Kinnear, 2013; Zak and Kosakowska, 2016). In addition, the biosynthesis of cyanobacterial secondary metabolites is estimated to consume a remarkable portion of metabolic energy, constituting a significant cost for the producing cell (Briand et al., 2012). However, natural environments are colonized by various clones producing different sets of metabolites (Briand et al., 2009). It has been then proposed that the environment may favor the selection of *Microcystis* clones that present the most-adapted metabolite composition (Welker et al., 2007; Martins et al., 2009; Agha and Quesada, 2014).

Recently, the development of modern mass spectrometry approaches has provided a new opportunity for describing the occurrence and the diversity of cyanobacterial metabolites (Yang et al., 2013; Briand et al., 2016a). In order to better understand the differences in metabolite production between clones of various bloom-forming cyanobacteria from different localities, we investigated here the clonal diversity of 24 *Microcystis* strains, originating from various geographical areas, using an innovative approach based on global molecular networking.

## Materials and Methods

### Sampling, Isolation and Cultivation of *Microcystis* Monoclonal Strains

The study was performed from 24 mono-clonal non-axenic cultures of *Microcystis* spp. maintained at 25°C in 15-mL vessels with Z8 media in the PMC (Paris Museum Collection) of living cyanobacteria^1^. Larger volume of all strains was simultaneously cultivated during one month in triplicates in 50 mL Erlenmeyer’s vessels at 25°C using a Z8 medium with a 16 h: 8 h light/dark cycle (60 μmol.m^-2^.s^-1^). All trains were then investigated for their MC production by Adda-microcystin AD4G2 ELISA kit (Abraxis). Cyanobacterial cells were centrifuged (at 4,000 g for 10 min), then freeze-dried and weighted, and stored at -80°C prior to DNA and metabolite analyses.

### DNA-Extraction, PCR, Sequencing, and Phylogenetic Analyses

DNA was extracted with Qiagen Kit (Cat N° 69506) according to manufacturer’s instructions. The presence and the quality of the extracted DNA was checked by observing the 260/280-nm ratio and the absorbance spectra between 200 and 800 nm using a NanoDrop spectrophotometer (SAFAS, Monaco). PCR reaction was performed with *mcyA* specific primers developed for *Microcystis* (*mcyA*_S AAAAACCCGCGCCCTTTTAC and *mcyA*_AS AGGCAGTTGGAGAATCACGG) in order to investigate the presence of this gene in the different strains. In parallel, the region containing a fragment 16S rRNA and another of the 16S-23S ITS was amplified using primer couples previously described in Gugger and Hoffmann (2004) and Iteman et al. (2000), respectively. The amplification was performed in a mix of 0.1 μL (100 μM) of each primer, 12.5 μL of MyTaq RedMix polymerase (Bioline^®^) and 2 μL (∼200 ng) of each DNA samples (25 μL final volume). The PCR product was sequenced (Genoscreen, France) using the same primers. The partial 16S and 16S-23S ITS sequences of all strains were deposited to GenBank (Accession numbers MH892877–MH892900 and MH899657–MH899680, respectively).

The *Microcystis* 16S-23S ITS gene sequences were compared to a selection of similar (>93% identity) sequences retrieved from NCBI according to nucleotide BLAST search (basic local alignment search tool). The different sequences were aligned with CodonCode Aligner and non-homologous regions of the sequence alignment were manually deleted with BioEdit tool (Version 7.2.5). The phylogeny of the aligned 16S-23S ITS sequences was performed using the MEGA V.6 software. The tree based on maximum likelihood (ML) was constructed with 1000-bootstrap replicates, performing a branch lengths iteration and global rearrangements.

### Metabolome Biomass Extraction and Analysis by Mass Spectrometry

The 20 mL of biomasses of the 24 *Microcystis* strain cultures were centrifuged (4,000 rpm, 10 min), the culture media discarded, and then freeze-dried. The lyophilized cells were weighted then sonicated 2 min in acetonitrile/methanol/water (40/40/20) acidified at 0.1% of formic acid with a constant ratio of 100 μL of solvent for 1 mg of dried biomass, centrifuged at 4°C (12,000 *g*; 5 min). Two micro liter of the supernatant were then analyzed on an UHPLC (Ultimate 3000, Thermo Fisher Scientific) coupled with a mass spectrometer (ESI-Qq-TOF Maxis II ETD, Bruker).

Ultra high performance liquid chromatography (UHPLC) was performed on 2 μL of each of the metabolite extracts using a Polar Advances II 2.5 pore C_18_ column (Thermo^®^) at a 300 μL.min^-1^ flow rate with a linear gradient of acetonitrile in 0.1% formic acid (5 to 90% in 21 min). The metabolite contents were analyzed in triplicate for each strain using an electrospray ionization hybrid quadrupole time-of-flight (ESI-QqTOF) high resolution mass spectrometer (Maxis II ETD, Bruker) on positive simple MS or on positive Collision Ion Dissociation (CID) autoMSMS mode with information dependent acquisition (IDA), on the 50–1500 *m/z* rang at 2 Hz or between 2 and 8 Hz speed, for MS and MS/MS, respectively, according to relative intensity of parent ions, in consecutive cycle times of 2.5 s, with an active exclusion of previously analyzed parents. The data were analyzed with the Data Analysis 4.4 and MetaboScape 3.0 software for internal recalibration (<0.5 ppm for each sample, as an internal calibrant of Na formate was injected at the beginning of each analysis), molecular feature search and MGF export. Peak lists were generated from MS spectra (between 1 and 15 min of the LC gradient), with a filtering of the noise fixed at the threshold of 0.1% of the maximal intensity, and combining all charge states and related isotopic forms. The annotation of the metabolite was attempted according to their molecular formula deduced from the precise mass and the isotopic pattern of each molecules and the presence of certain diagnostic ions, according to an in-house database of above 850 cyanobacteria metabolites (Supplementary Table S1; The csv export of the mass list containing respective molecular formula was searched for automatic identification using MetaboScape 3.0) and confirmed using GNPS molecular networking thank to their relative MS/MS fragmentation patterns and of few commercially available standard molecules analyzed similarly, in the same manner.

### Data and Statistical Analysis

Heatmap representation of the global metabolome of the 24 *Microcystis* spp. monoclonal strains was performed with Gene-E tool^2^ using the relative quantification (pic area) of 2051 molecular features analyzed on HR ESI-Qq-TOF using MetaboScape 3.0 (Bruker) with a >5000 counts and 400–2000 Da threshold, considering peak presents in at least three different trains and in at least six consecutive MS scans (S/N threshold value setup > 6). Then, the hierarchical clustering was performed according to Bray-Curtis distance method. NMDS and PERMANOVA analyses were performed using MicrobiomeAnalyst platform^3^ in order to investigate the influence of the species, the sampling localities and of the production of MCs, described as the variables, on the global metabolite distribution of the global metabolome observed on ESI-Qq-TOF for the 24 strains.

Using the whole MS/MS data (converted in .mgf format) obtained for the 24 strains taken together, a molecular network was produced using the online tool available at Global Natural Products Social molecular networking server (GNPS)^4^ (Yang et al., 2013). The data were clustered with MS-Cluster (1.0-Da parent mass tolerance and 0.5-Da MS/MS fragment ion tolerance). A network was then created (edges were filtered using the cosine-score > 0.6 and the more-than-five-matching-peak thresholds), without considering the respective retention times of the analytes. The spectra were automatically searched for annotation against the GNPS spectral libraries (thresholds fixed for score above 0.6 and at least five matched peaks). The different clusters of the network were attemptingly annotated by comparing in the spectra of each relative node their monoisotopic mass according to MS and MS/MS fragmentation pattern matches against our in-house cyanobacteria metabolite databases (Supplementary Table S1). Molecular networks were visualized using Cytoscape 3.2.1.

## Results

### Morphologic and Phylogenetic Characterization

**FIGURE 2:**
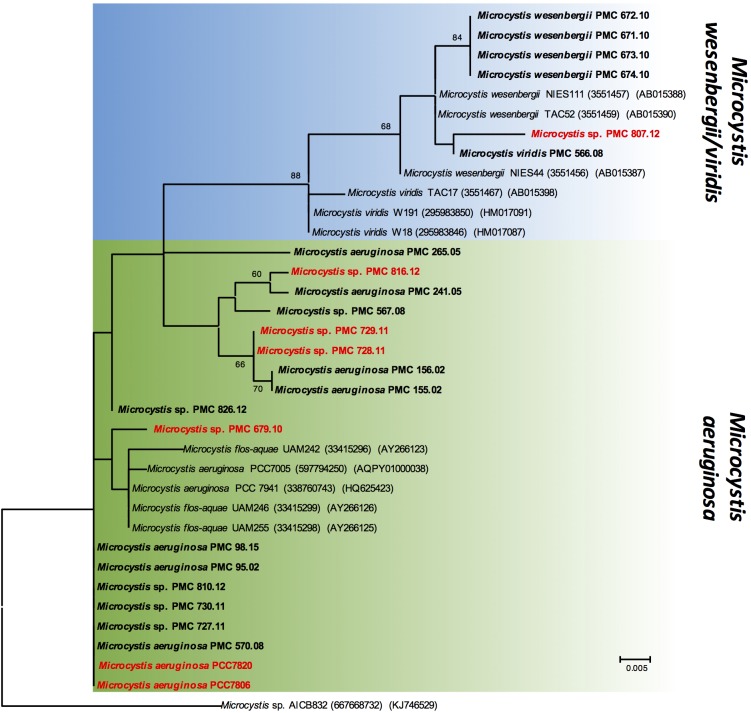
Maximum likelihood (ML) tree based on partial 16S-23S ITS sequences. The sequences obtained in this study are indicated in bold. The strains, which exhibit *mcyA* gene, are indicated in red. Other sequences were retrieved from GenBank, accession numbers in brackets. Bootstrap values > 60% are shown at the nodes. The scale bar indicates number of nucleotide substitutions per site. The *Microcystis* sp. AICB832 was used as an out-group.

The genetic relationships among the 24 *Microcystis* strains were first investigated by analyzing a 1380-bp 16S fragment (Table 1). This sequence comparison indicates that all *Microcystis* morpho-species are grouped in a unique and homogenous group, due to the high sequence conservation of this fragment (data not shown). Using 16S-23S ITS fragments (over 600 bp long), the phylogenetic analysis showed a clear distinction between *M. aeruginosa* and *M. wesenbergii/viridis* morpho-species (Figure 2). Interestingly, the different strains possessing the MC synthesis gene *mcyA* (indicated in red) did not cluster together on the phylogenetic tree, suggesting that the ability to produce MC constitutes a feature that is disconnected from strain phylogeny.

**Table 1:** List of *Microcystis* spp. strains used in this study, their area of origin, the ELISA MC screening, the *mcyA* gene presence and their respective 16S-ITS sequence Accession numbers.

Strain Name	Species	Country	Locality/area	MC ELISA detection	*mcyA* PCR detection	16S Accession number	16S-23S ITS Accession number
PCC 7806	*M. aeruginosa*	Netherlands	Braakman	+	+	MH892877	MH899657
PCC 7820	*M. aeruginosa*	Scotland	Balgavies	+	+	MH892878	MH899658
PMC 95.02^a^	*M. aeruginosa*	France	Villerest	-	-	MH892879	MH899659
PMC 98.15^a^	*M. aeruginosa*	France	Villerest	-	-	MH892880	MH899660
PMC 155.02	*M. aeruginosa*	Sénégal	Djoudj	-	-	MH892881	MH899661
PMC 156.02	*M. aeruginosa*	Sénégal	Djoudj	-	-	MH892882	MH899662
PMC 241.05	*M. aeruginosa*	Burkina Faso	Ouahigouya	+	+	MH892883	MH899663
PMC 265.06	*M. aeruginosa*	Burkina Faso	Sian	-	-	MH892884	MH899664
PMC 566.08^b^	*M. wesenbergii/viridis*	France	Varennes sur Seine	-	-	MH892885	MH899665
PMC 567.08^b^	*M. wesenbergii/viridis*	France	Varennes sur Seine	-	-	MH892886	MH899666
PMC 570.08	*M. aeruginosa*	France	Souppes sur Loing	-	-	MH892887	MH899667
PMC 671.10^c^	*M. wesenbergii/viridis*	France	Eure et Loire	-	-	MH892888	MH899668
PMC 672.10^c^	*M. wesenbergii/viridis*	France	Eure et Loire	-	-	MH892889	MH899669
PMC 673.10^c^	*M. wesenbergii/viridis*	France	Eure et Loire	-	-	MH892890	MH899670
PMC 674.10^c^	*M. wesenbergii/viridis*	France	Eure et Loire	-	-	MH892891	MH899671
PMC 679.10^c^	*M. aeruginosa*	France	Eure et Loire	+	+	MH892892	MH899672
PMC 727.11^d^	*M. aeruginosa*	France	Valence	-	-	MH892893	MH899673
PMC 728.11^d^	*M. aeruginosa*	France	Valence	+	+	MH892894	MH899674
PMC 729.11^d^	*M. aeruginosa*	France	Valence	+	+	MH892895	MH899675
PMC 730.11^d^	*M. aeruginosa*	France	Valence	-	-	MH892896	MH899676
PMC 807.12^e^	*M. wesenbergii/viridis*	France	Champs sur Marne	+	+	MH892897	MH899677
PMC 810.12^e^	*M. aeruginosa*	France	Champs sur Marne	-	-	MH892898	MH899678
PMC 816.12^e^	*M. aeruginosa*	France	Champs sur Marne	+	+	MH892899	MH899679
PMC 826.12^e^	*M. aeruginosa*	France	Champs sur Marne	-	-	MH892900	MH899680


*Stains isolated from the same sample collected the same day from different French area are indicated with: ^*a*^= Villerest (2008); ^*b*^= Varennes-sur-Seine (2008); ^*c*^= Eure et Loire (2010); ^*d*^= Valence (2011); ^*e*^= Champs-sur-Marne (2012).*

### Global Metabolome Analyses

**FIGURE 3:**
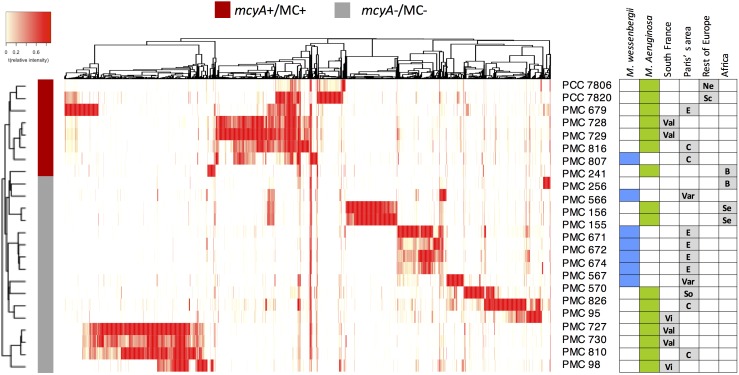
Heatmap representation of the metabolome of the 24 *Microcystis* spp. monoclonal strains analyzed using HR ESI-Qq-TOF, representing 2051 different analytes (in a 400–2000 Da window; present in at least three strains, with a signal to noise ratio in excess of 6, and respective relative peak intensity superior to 5000-count in at least one sample threshold) identified by MetaboScape software. The hierarchical clustering between strains was performed according to Bray-Curtis distance method. Green and blue squares indicate *M. aeruginosa* and *M. wesenbergii/viridis*, respectively. Sampling localities are: C, Champs-sur-Marne; B, Burkina Faso; E, Eure et Loire; Ne, Netherlands; Sc, Scotland; Se, Senegal; So, Souppes-sur-Loin; Var, Varennes-sur-Seine; Val, Valence; Vi, Villerest.

Metabolomic shotgun analyses reveal discriminant metabolic profiles among strains collected from both different or identical sites. While previous works highlighted the metabolic diversity of some *Microcystis* strains based on few identified metabolites or cyanotoxins (Welker et al., 2004, 2006; Martins et al., 2009), we present here a global picture of the metabolome of each strain (Figure 3 and Supplementary Figure S1). This representation clearly shows a clustering of all strains producing MCs (*mcyA*+/MC+, in red), on one side, and of other strains not producing MCs (*mcyA*-/MC-, in gray). This clustering shows that some strains from the same environment exhibit very similar metabolite fingerprints (e.g., PMC 728.11 and 729.11), while other strains from the same location exhibit much more dissimilar metabolite fingerprints (e.g., PMC 728.11 and 727.11), being more similar to strains from faraway locations (e.g., PMC 729.11 and 816.112). Additional non-metric multidimensional scaling (nMDS) and PERMANOVA analyses based on Bray-Curtis index indicated that the ability to produce MC seems to be the first main driver of the global metabolome of *Microcystis* molecular fingerprinting, while the species and the location represent less explicative parameters (Supplementary Figure S2).

### Metabolite Molecular Network

**FIGURE 4:**
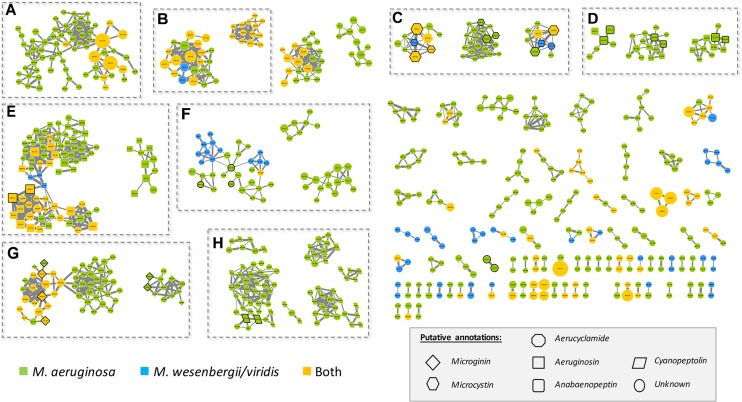
Molecular network generated from MS/MS spectra from the 24 *Microcystis* strains using the GNPS tool (all data and results are freely available at the address http://gnps.ucsd.edu/ProteoSAFe/status.jsp?task=c017414365e84334b38ae75728715552). The nodes of the analytes detected in *M. aeruginosa* or *M. wessenbergii/viridis* strains only are indicated in green and blue, respectively, when analytes detected in both species are indicated in orange. Uncharacterized analytes are indicated by circles constituting potential new analogs. Analytes whom individual masses match with known secondary metabolites from cyanobacteria (listed in Supplementary Table S1) are indicated by specific shapes as shown on picture caption. Standard molecules analyses similarly, are indicated by heavy black lines. Only cluster regrouping at least 2 analytes are represented. A, di- or tri-peptide cluster; B, Unknown metabolite clusters; C, microcystin clusters; D, anabaenopeptin clusters; E, aeruginosin clusters; F, aerucyclamide clusters; G, microginin clusters; H, cyanopeptolin clusters. The clusters on the right are corresponding to un-annotated and smaller clusters.

A molecular network was generated based on the global fragmentation pattern profile of all observed metabolites in the 24 strains investigated. The Global Natural Product Social network (GNPS) algorithm automatically compares all MS/MS spectra by aligning them one by one. This algorithm groups identical molecules (presenting identical mass and fragmentation patterns) and assigns a cosine score ranking from 0 to 1 to each alignment. From the present dataset, the resulting network is constituted of a total of 925 nodes from the 1374 different analytes which MS/MS data have been obtained (Supplementary Figure S1). It represents a starting point for the annotation of unidentified metabolites, according to respective cluster annotations (deduced from the presence of various annotated nodes from the same molecular structure family), and for the description of their occurrence in *M. aeruginosa* and/or *M. wesenbergii/viridis* strains (Figure 4).

*Microcystis* strains produce a large set of chemically diverse metabolites, of which the principal clusters can be identified thanks to analytical standards available for some cyanobacterial secondary metabolite families, or to match with components from publicly available libraries from the GNPS platform, such as HMDB, NIST14, or METLIN. More than 25% of the strains producing these analytes appear to be specific to *M. wesenbergii/viridis* strains, more than 50% are specific to *M. aeruginosa*, and the rest being observed in both species.

Different analytes were grouped in the same molecular clusters based on the similarity of their fragmentation patterns, with each cluster being potentially specific to the structure of the chemical families. Among those larger clusters, we were able to annotate some that were constituted by ions of small metabolites, such as di- and tri-peptides (1 cluster in A area), of microcystins (3 clusters in C area), of anabaenopeptins (3 clusters in D area), of aeruginosins (2 clusters in E area), of aerucyclamides (3 clusters in F area), of microginins (2 clusters in H area), and cyanopeptolins (6 clusters in I area), together with various clusters of unknown components, comprising non-identified ions (for example 2 clusters in B area). Less than a third of the metabolites observed here could be annotated by their respective mass and fragmentation patterns when compared to those of the more than 850 metabolites of freshwater cyanobacteria described so far and listed in Supplementary Table S1. These un-identified ions that belong to annotated clusters are then considered as potential new analogs of their respective molecular family.

### Known Cyanobacteria Secondary Metabolite Clusters

#### Microcystins

**FIGURE 5:**
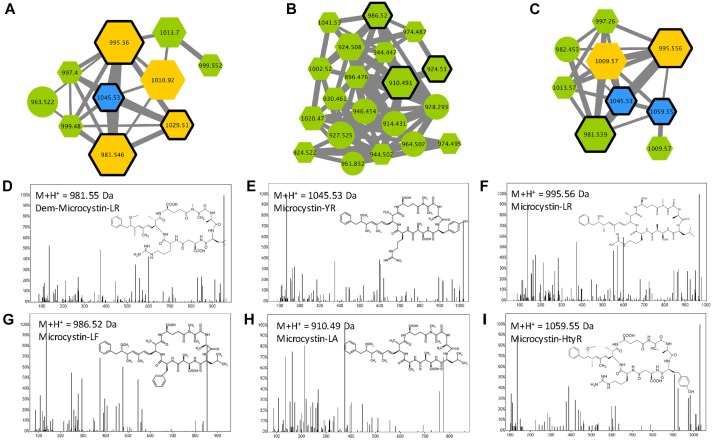
Microcystin clusters **(A–C)** highlighted by the GNPS analysis based on the MS/MS CID fragmentation spectra obtained from the 24 *Microcystis* strains. Analytes detected in *M. aeruginosa* or *M. wessenbergii/viridis* strains only are indicated in green and blue, respectively, when analytes detected in both species are in orange. Analytes whom individual masses match with known microcystins (MCs) are indicated by hexagons. Standard molecules analyses similarly, are indicated by heavy black lines. Example of MS/MS spectra and chemical structures are shown for (Asp_3_)-microcystin-LR **(D)**, microcystin-YR **(E)**, -LR **(F)**, -LF **(G)**, -LA **(H)** and -HtyR **(I)**. Notice that (M+H)^+^ and (M+2H)^2+^ ions may be grouped in distinct clusters (**A–C**, respectively).

Microcystins are cyclic heptapeptides that were first described in *M. aeruginosa*. More than 250 different variants have been described so far (Catherine et al., 2017); 138 are references in our database for cyanobacterial metabolites (Supplementary Table S1). They are characterized by the presence of a non-proteinaceous amino acid in position 5 (Adda), two amino acids derived from Asp and Glu in position 3 and 6, respectively, and 2 very variable positions (2 and 4), that serve as reference to the name of the variant. Three microcystin clusters were highlighted according to the presence of six standard molecules (Dmet(Asp3)-MC-LR, MC-LR, MC-YR, MC-LA, MC-LF, and MC-HtyR) analyses in parallel of the 24 *Microcystis* extracts with the same protocol (Figure 5). Other components of these clusters correspond to ions presenting a match of their respective mass with those of other previously described MC variants (Supplementary Table S1), or for 18% of them, to potentially new analogs. Observation of their respective MS/MS spectra showed that these metabolites present distinct but similar fragmentation patterns to those of other known MC variants.

#### Aeruginosins

**FIGURE 6:**
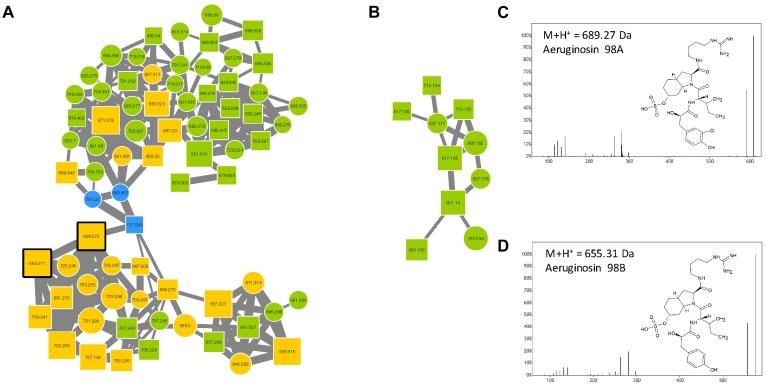
Aeruginosin clusters **(A,B)** highlighted by the GNPS analysis based on the MS/MS CID fragmentation spectra obtained from the 24 *Microcystis* strains. Analytes detected in *M. aeruginosa* or *M. wessenbergii/viridis* strains only are indicated in green and blue, respectively, when analytes detected in both species are in orange. Analytes whom individual masses match with known aeruginosins are indicated by squares with right corners. Standard molecules analyses similarly, are indicated by heavy black lines. Example of MS/MS spectra and chemical structures are shown for aeruginosin 98A **(C)** and 98B **(D)**.

Aeruginosins constitute a family of linear tetrapeptides that were first described in *M. aeruginosa*, and that represent more than 94 different variants that have been described so far (Supplementary Table S1). Their MS/MS fragmentation patterns are often characterized by the presence of a Choi fragment (immonium with 140.109 *m/z*) and other recurrent fragments from Hpla or Pla. Their composition is rather variable and the members of this family exhibit masses between 430 and 900 Da (Welker and Von Döhren, 2006). The molecular network obtained from the 24 *Microcystis* strains exhibits two aeruginosin clusters (Figure 6) that were highlighted by to the presence of two standard molecules (aeruginosin 98A and 98B). The other components of these clusters correspond to ions with masses that match previously described variants of aeruginosin (Supplementary Table S1), or for 47% of these compounds, to potentially new analogs.

#### Anabaenopeptins

**FIGURE 7:**
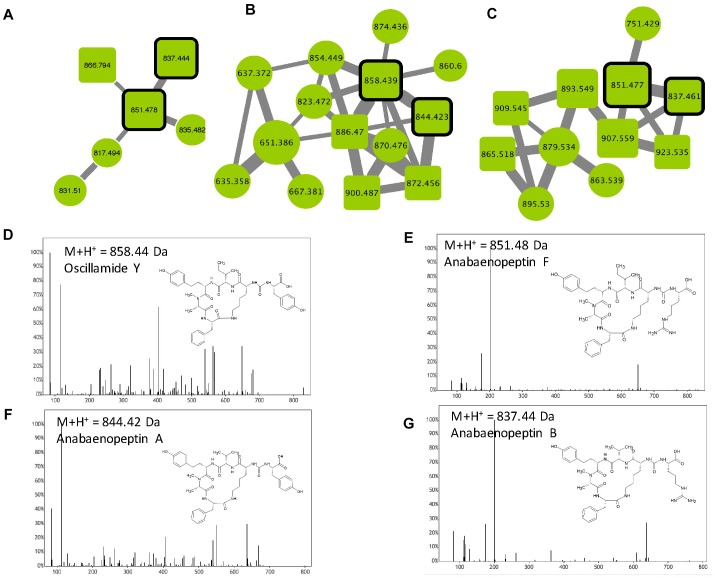
Anabaenopeptin clusters **(A–C)** highlighted by the GNPS analysis based on the MS/MS CID fragmentation spectra obtained from the 24 *Microcystis* strains. Analytes detected in *M. aeruginosa* strains are indicated in green. Analytes whom individual masses match with known anabaenopeptins are indicated by squares with rounded corners. Standard molecules are indicated by heavy black lines. Uncharacterized analytes are indicated by circles constituting potential new analogs. Example of MS/MS spectra and chemical structures are shown for Oscillamide Y **(D)**, anabaenopeptin F **(E)**, A **(F)** and B **(G)**. Notice that (M+H)^+^ and (M+2H)^2+^ ions may be represented by different nodes grouped in distinct clusters (**A–C**, respectively).

Anabaenopeptins constitute a very diverse family of cyclic hexapeptides that have been described so far in *Microcystis, Planktothrix, Anabaena, Aphanizomenon*, and *Nostoc*. Over 75 different variants have been described to date (Supplementary Table S1). These compounds are characterized by the presence of a peptide bond between the D-Lys in position 2 and the carboxylic group of the amino acid in position 6. Except for the D-Lys (position 2), all other positions are variable, allowing a large structural diversity of the family with members exhibiting masses between 750 and 950 Da (Welker and Von Döhren, 2006). Three anabaenopeptin clusters were highlighted in this study (Figure 7) according to the presence of 4 standard molecules (anabaenopeptins A, B, and F, and oscyllamide Y). Other components of these clusters correspond to ions presenting a match with the mass of other previously described variants (Supplementary Table S1), or for 57% of them, to compounds that very likely correspond to potentially new analogs. All observed anabaenopeptin compounds are from *M. aeruginosa* strains, suggesting that *M. wesenbergii/viridis* strains are not capable of synthesizing molecules of this family and may not possess the corresponding *apt* synthetic gene cluster.

#### Cyanopeptolins

**FIGURE 8:**
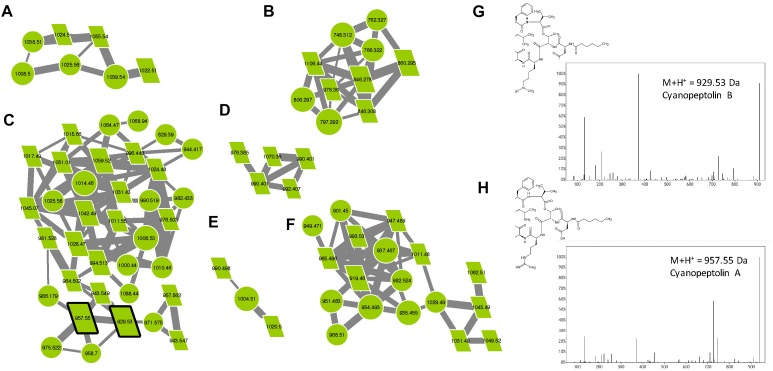
Cyanopeptolin clusters **(A–F)** highlighted by the GNPS analysis based on the MS/MS CID fragmentation spectra obtained from the 24 *Microcystis* strains. Analytes detected in *M. aeruginosa* strains are indicated in green. Analytes whom individual masses match with known cyanopeptolin are indicated by parallelepipeds. Standard molecules are indicated by heavy black lines. Uncharacterized analytes analyses similarly, are indicated by circles constituting potential new analogs. Example of MS/MS spectra and chemical structures are shown for cyanopeptolin B **(G)** and A **(H)**.

Cyanopeptolins belong to a large family of cyclic depsipeptides that also contains micropeptins and aeruginopeptins, representing over 170 variants. Those molecules are characterized by the presence of the non-proteinaceous amino acid Ahp and by a six-aa-long ring formed by an ester bound between Thr or Pro in position 1 and the carboxylic group of the N-terminal amino acid (position 6). The lateral chain exhibits variable length and is constituted by one or two amino acids and is potentially linked to an aliphatic fatty acid (Welker and Von Döhren, 2006). In the molecular network, two analytes of the cyanopeptolin clusters correspond to two standard molecules (cyanopeptolin A and B), and various other components correspond to ions presenting a mass that corresponds to those of different previously described variants (Supplementary Table S1), allowing us to annotate them as cyanopeptolin-specific clusters (Figure 8). Over 47% of the analytes present in these clusters correspond to unknown compounds representing potentially new analogs. We observe here that all these cyanopeptolin compounds are from *M. aeruginosa* strains, suggesting that *M. wesenbergii/viridis* strains are not capable of synthesizing molecules of this family and may not possess the corresponding *mcn/oci* synthetic gene cluster either.

#### Microginins

**FIGURE 9:**
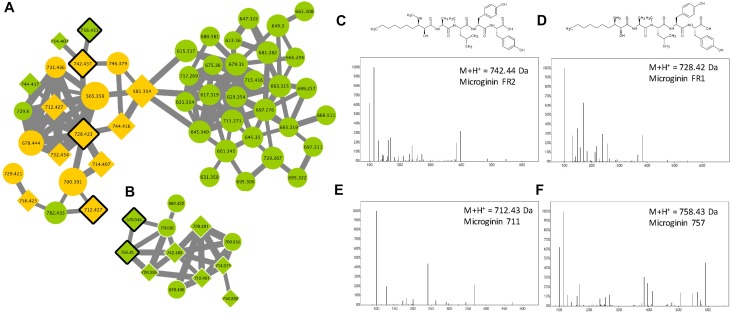
Microginin clusters **(A,B)** highlighted by the GNPS analysis based on the MS/MS CID fragmentation spectra obtained from the 24 *Microcystis* strains. Analytes detected in *M. aeruginosa* strains only are indicated in green and blue, when analytes detected in both species are in orange. Analytes whom individual masses match with known microginin are indicated by 45°-tilted squares. Standard molecules analyses similarly, are indicated by heavy black lines. Uncharacterized analytes are indicated by circles constituting potential new analogs. Example of MS/MS spectra and chemical structures are shown for microginin FR2 **(C)**, FR1 **(D)**, 711 **(E)** and 757 **(F)**. Notice that spectra of microginin BN578 and SD755 (are not shown here).

Microginins are linear pentapeptides (the length of the sequence varies from 4 to 6 amino acids) initially identified from *M. aeruginosa*, then from other species and in other genera such as *Planktothrix* (Welker and Von Döhren, 2006). These molecules are composed of a characteristic non-proteinaceous amino acid Ahda at their N-terminus, with the other position bearing variable amino acid structures, comprising Tyr, Pro Hty, Trp, Ala, Ser, or others. Relatively few microginin variants (less than 40) have been described so far (Supplementary Table S1). According to the molecular network generated in this study (Figure 9), over 67% of the analytes present in the two microginin clusters correspond to unknown compounds constituting potentially new analogs, when six standard molecules could have been retrieved from the present analysis (microginin 757, 711, BN578, FR1, FR2, and SD755).

#### Aerucyclamides/Cyanobactins

**FIGURE 10:**
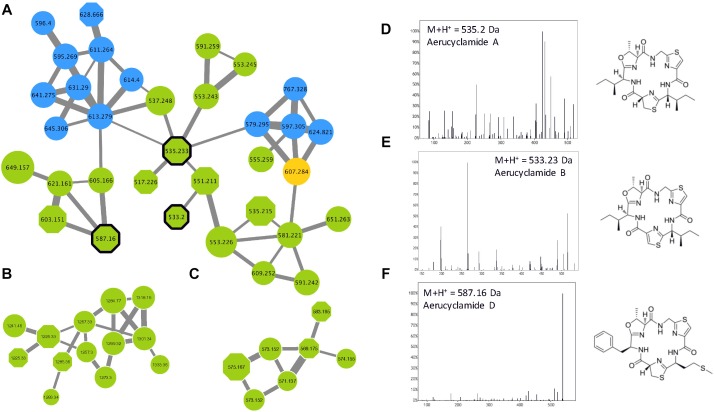
Aerucyclamide clusters **(A–C)** highlighted by the GNPS analysis based on the MS/MS CID fragmentation spectra obtained from the 24 *Microcystis* strains. Analytes detected in *M. aeruginosa* or *M. wessenbergii/viridis* strains only are indicated in green and blue, respectively, when analytes detected in both species are in orange. Analytes whom individual masses match with known aerucyclamides are indicated by octagons. Uncharacterized analytes are indicated by circles constituting potential new analogs. Standard molecules analyses similarly, are indicated by heavy black lines. Example of MS/MS spectra and chemical structures are shown for aerucyclamide A **(D)**, B **(E)** and D **(F)**.

The name “cyanobactin” has been proposed to group all cyclic peptides containing proteinogenous amino acids that are post-translationally modified in heterocyclic amino acids and isoprenoid derivatives (Sivonen et al., 2010). It comprises various cyclamides (cyclic peptides of 6 amino acids) that have been identified in freshwater cyanobacteria such as *Microcystis, Planktothrix*, and *Nostoc*, but also in symbiotic cyanobacteria species. More than 30 variants have been described so far, but more molecules could be related to the family that represent a very large variety of chemical structures (Martins and Vasconcelos, 2015). Three different cyanobactin clusters were observed in the molecular network (Figure 10), comprising three aerucyclamide standard molecules (aerucyclamide A, B, and D) that were identified by GNPS tools. More than 75% of the analytes from these clusters represent potentially new analogs that need to be characterized by further dedicated analyses.

### Uncharacterized Cyanobacteria Metabolite Clusters

#### Potential Primary Metabolite Clusters

Various other important clusters contained some unknown analytes that are mostly present in high amounts (higher peak intensity shown by larger forms) and in a large set of strains from both *M. aeruginosa* and *M. wesenbergii/viridis* (Figure 4). These compounds exhibit molecular masses between 300 and 600 Da, suggesting that they could correspond to relatively small molecules (Supplementary Figures S3, S4). We speculate that these components could correspond to primary metabolites used for the general metabolism of various strains, however, additional efforts should be made to annotate these ubiquitous molecules present in these specific clusters. Interestingly, the compounds from this latter cluster present high fragmentation patterns, illustrated by various similarity and high cosine scores (numerous and tick links between the different nodes of the clusters), suggesting they should present very similar structures.

#### Uncharacterized Secondary Metabolite Clusters

Interestingly, other clusters correspond to unknown components, some of them being only synthesized by *M. wesenbergii/viridis* as shown in Supplementary Figure S5. These compounds with relatively high molecular masses (between 950 and 1300 Da) might correspond to specific secondary metabolite families belonging to other known, but poorly characterized, families of cyanobacterial metabolites, such as microviridins and/or aeruginoguanidins, for which only few variants have been characterized and no standard molecules are available so far. Alternatively, they may correspond to a completely new family of metabolites, the existence of which was suggested by observing orphan NRPS/PKS clusters within various *Microcystis* genomes (Humbert et al., 2013).

Although the present MS/MS-based metabolomic approach was focused on the most intense analytes present in the biomass of the *Microcystis* cultures, we cannot fully exclude the possibility that some of these molecules could be produced by the small fraction of heterotrophic bacteria that colonized these non-axenic cultures.

## Discussion

### Biogeography and Genetic Diversity Attempt on *Microcystis* Strains

The phylogeny of the various *Microcystis* species is still under investigation. Morphological criteria comprising the form and size of the colony, the presence and the structure of the mucilage, the cell diameter, the organization of cells within colonies, together with the pigment content and some life cycle parameters were used to discriminate among different morpho-species (Komárek, 2016). Accordingly, five main *Microcystis* morpho-species (*M. aeruginosa, M. ichthyoblabe, M. viridis, M. novacekii, M. wesenbergii*) were proposed by Otsuka et al. (2001). However, with the recent development of different genetic and biochemical molecular markers, some results were contradictory to previous *Microcystis* taxonomy results. For instance, analysis based on 16S rRNA sequence alone revealed no differences among all these morpho-species (Otsuka et al., 2000). Considering both morphological and molecular markers, *Microcystis* seems to be classifiable into three groups: a smaller cell-size group comprising of *M. ichthyoblabe* and *M. flos-aquae*, a middle cell-size group composed of *M. aeruginosa* (incl. *M. novacekii*), and a larger cell-size group based on *M. wesenbergii* (Whitton, 2012). Although the size criteria classification seems practical, size varies according to the physiological state of the cells and is not tightly correlated with genetic markers.

According to the phylogenetic reconstruction based on the 16S-16S/23S ITS fragment that is in accordance with previous observations (Otsuka et al., 1999), the studied *Microcystis* strains were roughly divided into two groups: the *M. wesenbergii/viridis* group and the *M. aeruginosa* group (according to the size of the colonies observed under microscopes). In addition, the *mcyA*+ and *mcyA*- strains were broadly dispersed in both groups. These observations confirmed that the presence of *mcy* does not simply capitulate the phylogeny of *Microcystis* strains (Tillett et al., 2001). Therefore, the toxicity potential, through the MC synthesis, of each strain should be assessed regardless of its phylogenetic position.

Despite the very high similarity of *Microcystis* 16S rRNA sequences (>99.5%), low synteny and large genomic heterogeneity have been retrieved from investigating *Microcystis* genomes, deciphering a large cryptic diversity from various strains collected from different sites and continents (Steffen et al., 2012; Humbert et al., 2013). At another large geographic scale, genetic comparison of various *Microcystis* strains isolated from different Asian and European lakes based on 16S-ITS fragments did not show a location-specific clustering effect, indicating that the genetic distance between different genotypes from the same lake can be greater than between strains from very distanced environments (Humbert et al., 2005; Haande et al., 2007). Interestingly, large genomic islands have been detected in *Microcystis* genomes and metagenomes, and these mobile elements seem to greatly contribute to the clonal diversity within this genus (Steffen et al., 2012).

Our data on the molecular heterogeneity observed by metabolic fingerprinting between some strains originating from the same site (for example between PMC 810.12 and PMC 816.12, Champs-sur-Marne, France) also support this hypothesis. Interestingly, the fact that some *Microcystis* genotypes or chemotypes could be spread worldwide was also previously observed for some freshwater bacterioplankton species (Zwart et al., 1998), suggesting that these organisms may present peculiar physiological and/or large dispersal capabilities contributing to their successful colonization of a wide range of freshwater environments.

### MC-Producing Versus Non-MC Producing Metabolic Pattern of *Microcystis* Strains

Metabolome diversity has been also used as a molecular characteristic to help discriminate among various chemotypes (Ivanisevic et al., 2011), in addition to classical genotyping approaches that sometimes lack reliable characteristics for phylogenetic relationship discrimination. Few analytical methods have been investigated for chemo-taxonomic characterization of cyanobacteria strains or cells (Welker et al., 2006), according for example, to their fatty acid compositions (Gugger et al., 2002), or more recently to ribosomal proteins globally analyzed by MALDI-TOF on *M. aeruginosa* (Sun et al., 2016). Interestingly, this latter chemotaxonomic approach was able to group all the MC-producing strains in two distinct clades when the non-MC-producing strains were segregated in three other distinct clades. In a previous work, Martins et al. (2009) analyzed the metabolite diversity of various *M. aeruginosa* strains from Portuguese water supplies using MALDI-TOF MS and were able to observe MC production in almost half of the strains investigated. These observations also illustrate the fact that MC-producing clones can subsist in various environments, despite the important energetic cost required for MC gene cluster replication and the translation of its mega-enzyme complex (Briand et al., 2012). The biological advantage of producing MCs for some clones still remains a mystery, as the functional role of MC is still uncharacterized (Gan et al., 2012; Agha and Quesada, 2014).

In a similar manner, our molecular fingerprint approach based on global metabolome profiling using ESI-Qq-TOF discriminated clearly MC-producing strains from others. Taken together, these observations suggest that shotgun mass spectrometry chemotyping of cyanobacteria could constitute a promising tool for characterizing rapid biomarkers for toxicological assessment of strains isolated from the field. MC production could constitute a singular feature, that could constitute one of the key drivers of the global metabolic diversity of *Microcystis* strains, suggesting that MC could play a keystone function in cyanobacterial metabolite production. Indeed, it was previously hypothesized according to metabolomic observations that the characteristic of MC production could be compensated for in strains not producing MCs by the production of other metabolites, such as aeruginosamines (Martins et al., 2009; Pancrace et al., 2018) for unknown biological reasons (Tonk et al., 2009; Briand et al., 2016a). Such secondary metabolic compensatory processes, between and within the different peptide classes, have been previously suspected for both *Microcystis* (Martins et al., 2009; Pancrace et al., 2018) and *Planktothrix* (Tonk et al., 2005) in response to various growth conditions. However, further investigations with wider sampling are now required in order to increase the dataset that could better help test such metabolite functional hypothesis.

### Secondary Metabolite Diversity Within Known Metabolite Families

The molecular cluster identified by GNPS approach (Yang et al., 2013) can be annotated to match with spectral databases or the presence of standard molecules. In our hands, the global molecular networks obtained from the MS/MS dataset of the 24 strains examined in this study presents various clusters that have been annotated. Most of them correspond to main cyanobacterial metabolite families (Figure 4). We assumed that the nodes of these clusters with similar molecular masses to already known cyanobacterial metabolites very likely correspond to these specific metabolites, or alternatively, to isobaric analogs. In addition, all other nodes from those clusters that do not correspond to either standard or known analogs could be considered potentially new analogs that may correspond to new variants that remain to be characterized. These observations are in accordance with results of previous research indicating that different *Microcystis* strains can produce such various known and unknown secondary metabolites, according to both genetic or targeted metabolome analyses (Welker et al., 2004, 2006; Martins et al., 2009; Humbert et al., 2013). Surprisingly, few metabolite families such as aeruginoguanidine were not successfully detected in our GNPS analysis. Indeed, very few variants belonging to this family have been described (Supplementary Table S1) and their MS/MS fragmentation pattern has not been deeply characterized so far. In addition, the lack of available standard molecules and of knowledge of their respective fragmentation patterns makes aeruginosamines especially challenging to annotate with a GNPS-based approach.

However, our analyses revealed the large molecular diversity of *Microcystis* metabolites, according to the various new variants of known cyanobacterial metabolite families that remain to be characterized. The observation of various uncharacterized clusters also suggest that new metabolite families are still waiting to be discovered and described from this genus, and that further efforts to this end are still required. So far, *Microcystis* represents one of the most studied genera for its production of various metabolite families. However, the biological functions played by these molecules remain enigmatic and their growing molecular diversity revealed by global approaches, such as GNPS global metabolomic investigation or genomics, constitutes one of the questioning paradoxes in the field of microbiological evolution and diversity.

### Unknown Metabolite Families

Although the strains used in this study were not cultured in hyper-stringent axenic conditions, no noticeable contamination by fungi or heterotrophic bacteria were detected during the systematic screening of all strains under light microscope prior to the experiment. In addition, a previous metabolome analysis in PCC 7806 grown under axenic or non-axenic conditions did not detect any significant variation in the metabolites produced by the cyanobacteria (Briand et al., 2016b). We assume that the metabolite profiles observed here for the 24 strains are characteristic of the cyanobacteria and that the different metabolites observed in this study, including the unknown metabolite clusters highlighted by the network analysis, are genuinely produced by the cyanobacteria.

The non-annotated cluster observed in our GNPS analysis can potentially correspond to novel variants of known cyanotoxins or to a completely new family of cyanobacteria metabolites. Indeed, Humbert et al. (2013) showed that the genomes of ten *Microcystis* strains exhibit at least three orphan clusters with a specific NRPS/PKS signature that likely synthesize a yet-undescribed metabolite family. The unknown clusters we observed using the GNPS approach may correspond to these novel metabolite families, and structural elucidation of an expanding number of novel metabolites revealed by molecular networking is currently being performed for various cyanobacteria (Boudreau et al., 2015).

## Conclusion

In the present study, a comparison of the specific chemical footprints of 24 clonal *Microcystis* strains generated through modern ESI-qTOF mass spectrometry shows a global influence of microcystin production on metabolite content, rather than on their respective genotypes or sampling locality origins. The GNPS network of all metabolites highlighted the production of a wide set of chemically diverse metabolites, among which there were a few microcystins, and also many aeruginosins, microginins, cyanopeptolins, and anabaenopeptins, along with a large set of unknown molecules that remain to be characterized.

Innovative approaches based on shotgun metabolomic analyses using high-resolution mass spectrometry, such as those performed in this study, seem to provide a large variety of information on cyanobacterial chemical diversity, relevant to evolutionary, ecological, and toxicological purposes. This represents an interesting and relatively easy-to-perform alternative to genome sequencing for metabolite and/or toxic potential descriptions of cyanobacterial strains.

Global molecular network analysis also allows the depiction of the chemical diversity of the *Microcystis* metabolome in an interesting manner. More than half of the analytes described in the global molecular network were found to correspond to metabolites belonging to potentially new variants of known families or family members (presenting original fragmentation patterns) that are yet to be described at the structural and toxicological/bioactivity levels.

## Author Contributions

SM, ME, AC, CB, and BM conceived and designed the experiments. CD isolated all new strains of the PMC. CD, SM, AM, and CDJ performed the analysis. SM, CD, AM, and BM treated the data. All authors wrote and reviewed the manuscript.

## Conflict of Interest Statement

The authors declare that the research was conducted in the absence of any commercial or financial relationships that could be construed as a potential conflict of interest.

## Footnotes

1 http://www.mnhn.fr/fr/collections/ensembles-collections/ressources-biologiques-cellules-vivantes-cryoconservees/microalgues-cyanobacteries

2 https://software.broadinstitute.org/GENE-E/

3 http://www.microbiomeanalyst.ca/

4 http://gnps.ucsd.edu

## Supplementary Material

FIGURE S1 Representation of the analytes from the 24 *Microcystis* strains analyzed by MS simple and MS/MS positive mode, exhibiting the good representativeness of analytes selected for MS/MS analyses. All analyzed ions are represented according to their respective retention time and *m/z* ratio. For MS/MS data, the size of the circle being representative of their maximum peak intensity.

FIGURE S2 NMDS analysis of global metabolite patterns of the 24 *Microcystis* spp. monoclonal strains analyzed using HR ESI-TOF, with PERMANOVA analyses performed on MicrobiomeAnalyst platform with Bray-Curtis index according to the MC production **(A)** and to the genera **(B)**. “MC production,” “species,” and “locality” factor present significant impact on the global metabolome.

FIGURE S3 Unknown cluster “1” **(A)** highlighted by the GNPS analysis based on the MS/MS CID fragmentation spectra obtained from the 24 *Microcystis* strains. This cluster of uncharacterized molecules that present high fragmentation similarity **(B–G)** may correspond to a new family of metabolites that still need to be characterized. Analytes detected in *M. aeruginosa* or *M. wessenbergii/viridis* strains only are indicated in green and blue, respectively, when analytes detected in both species are in orange.

FIGURE S4 Unknown cluster “2” **(A)** highlighted by the GNPS analysis based on the MS/MS CID fragmentation spectra obtained from the 24 *Microcystis* strains. This cluster of uncharacterized molecules that present high fragmentation similarity **(B–E)** may correspond to a new family of metabolites that still need to be characterized. Analytes detected in *M. aeruginosa* and *M. wessenbergii/viridis* strains are indicated in orange.

FIGURE S5 Unknown cluster “3–5” **(A–C)** highlighted by the GNPS analysis based on the MS/MS CID fragmentation spectra obtained from the 24 *Microcystis* strains. These clusters of uncharacterized molecules that present, respectively, intrinsic high fragmentation similarity (examples of representative spectra are provided **D–F**) may correspond to families of metabolites that still need to be characterized. Analytes detected in *M. wessenbergii/viridis* strains only are indicated in blue.

TABLE S1 List of 852 cyanobacterial metabolites retrieved from the literature.
