# Supplementary figures and images for: Global Metabolomic Characterizations of Microcystis spp. Highlights Clonal Diversity in Natural Bloom-Forming Populations and Expands Metabolite Structural Diversity
Source: Front Microbiol. 2019 Apr 16;10:791. doi: 10.3389/fmicb.2019.00791 (PMC6477967; doi:10.3389/fmicb.2019.00791)

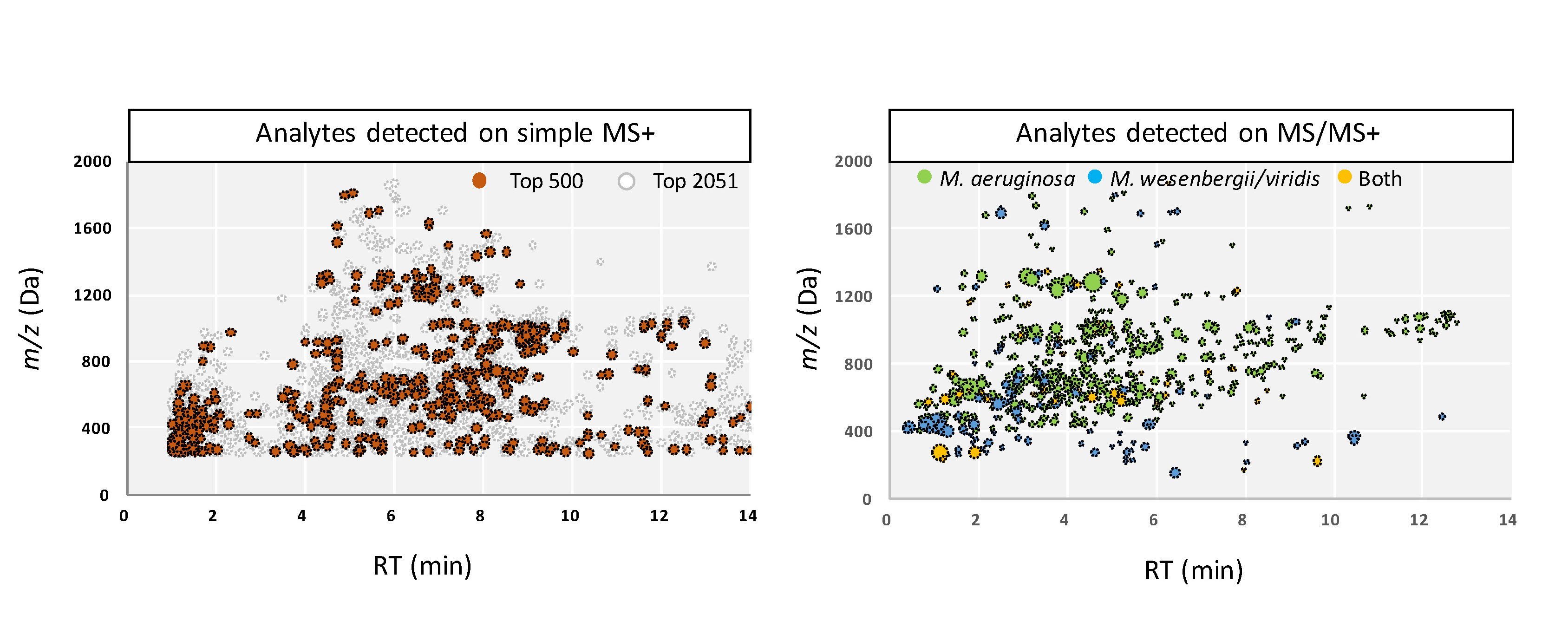

Supplement: FIGURE S1 — Representation of the analytes from the 24 Microcystis strains analyzed by MS simple and MS/MS positive mode, exhibiting the good representativeness of analytes selected for MS/MS analyses. All analyzed ions are represented according to their respective retention time and m/z ratio. For MS/MS data, the size of the circle being representative of their maximum peak intensity. [file Image_1.JPEG]

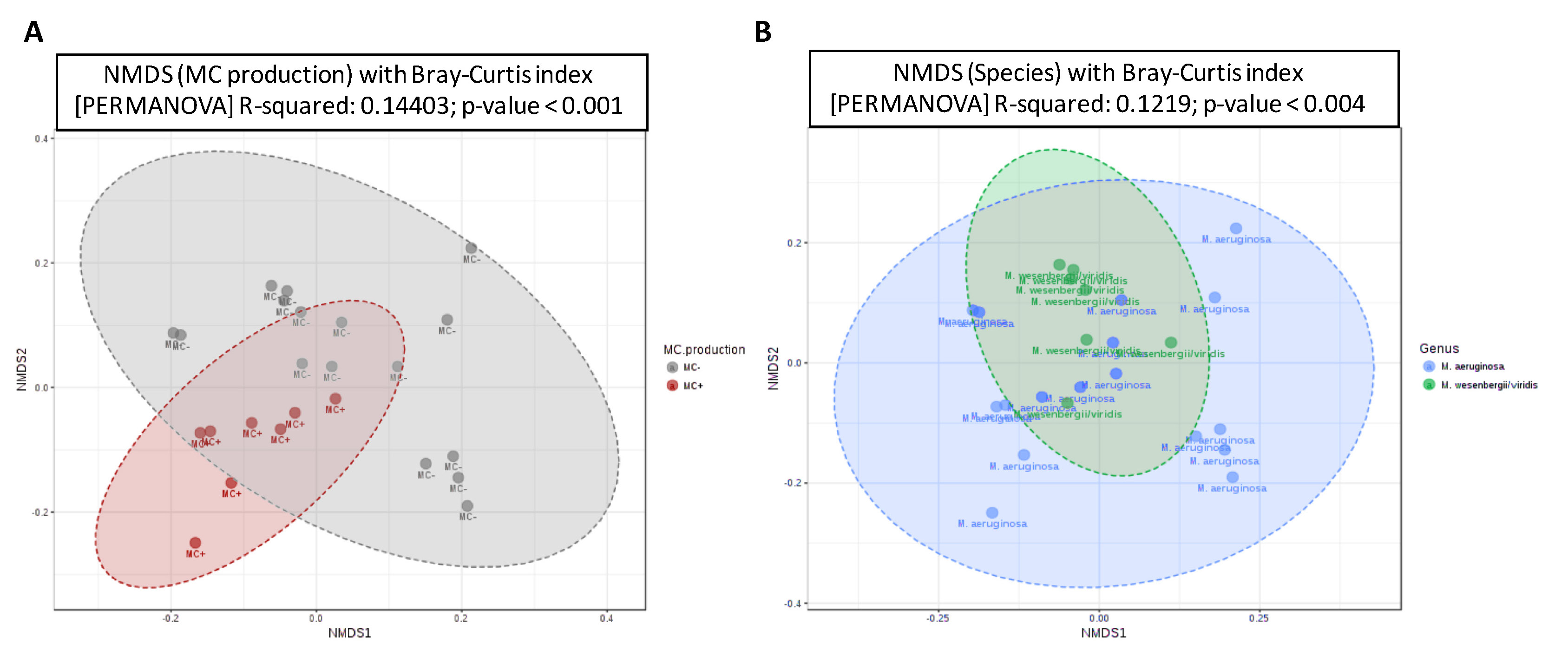

Supplement: FIGURE S2 — NMDS analysis of global metabolite patterns of the 24 Microcystis spp. monoclonal strains analyzed using HR ESI-TOF, with PERMANOVA analyses performed on MicrobiomeAnalyst platform with Bray-Curtis index according to the MC production (A) and to the genera (B). “MC production,” “species,” and “locality” factor present significant impact on the global metabolome. [file Image_2.JPEG]

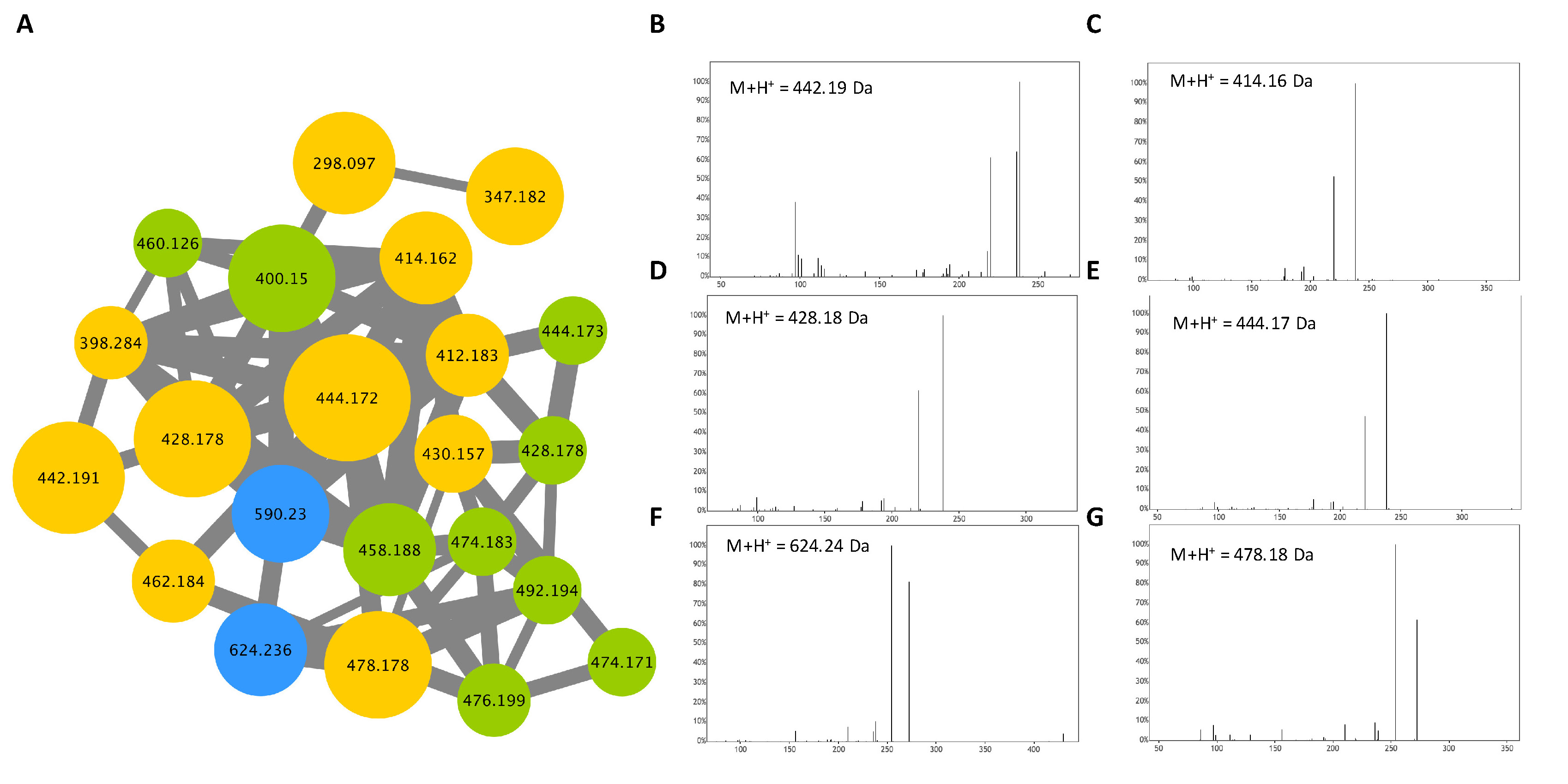

Supplement: FIGURE S3 — Unknown cluster “1” (A) highlighted by the GNPS analysis based on the MS/MS CID fragmentation spectra obtained from the 24 Microcystis strains. This cluster of uncharacterized molecules that present high fragmentation similarity (B–G) may correspond to a new family of metabolites that still need to be characterized. Analytes detected in M. aeruginosa or M. wessenbergii/viridis strains only are indicated in green and blue, respectively, when analytes detected in both species are in orange. [file Image_3.JPEG]

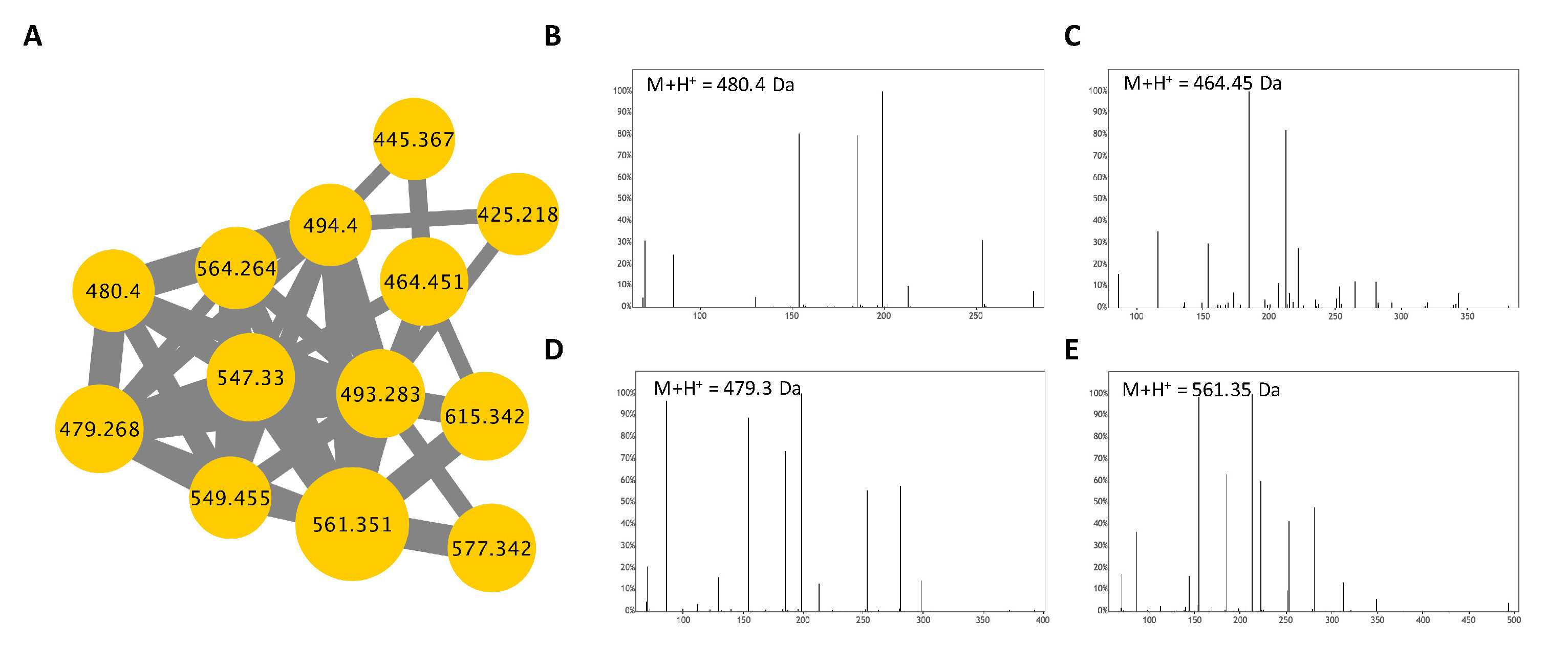

Supplement: FIGURE S4 — Unknown cluster “2” (A) highlighted by the GNPS analysis based on the MS/MS CID fragmentation spectra obtained from the 24 Microcystis strains. This cluster of uncharacterized molecules that present high fragmentation similarity (B–E) may correspond to a new family of metabolites that still need to be characterized. Analytes detected in M. aeruginosa and M. wessenbergii/viridis strains are indicated in orange. [file Image_4.JPEG]

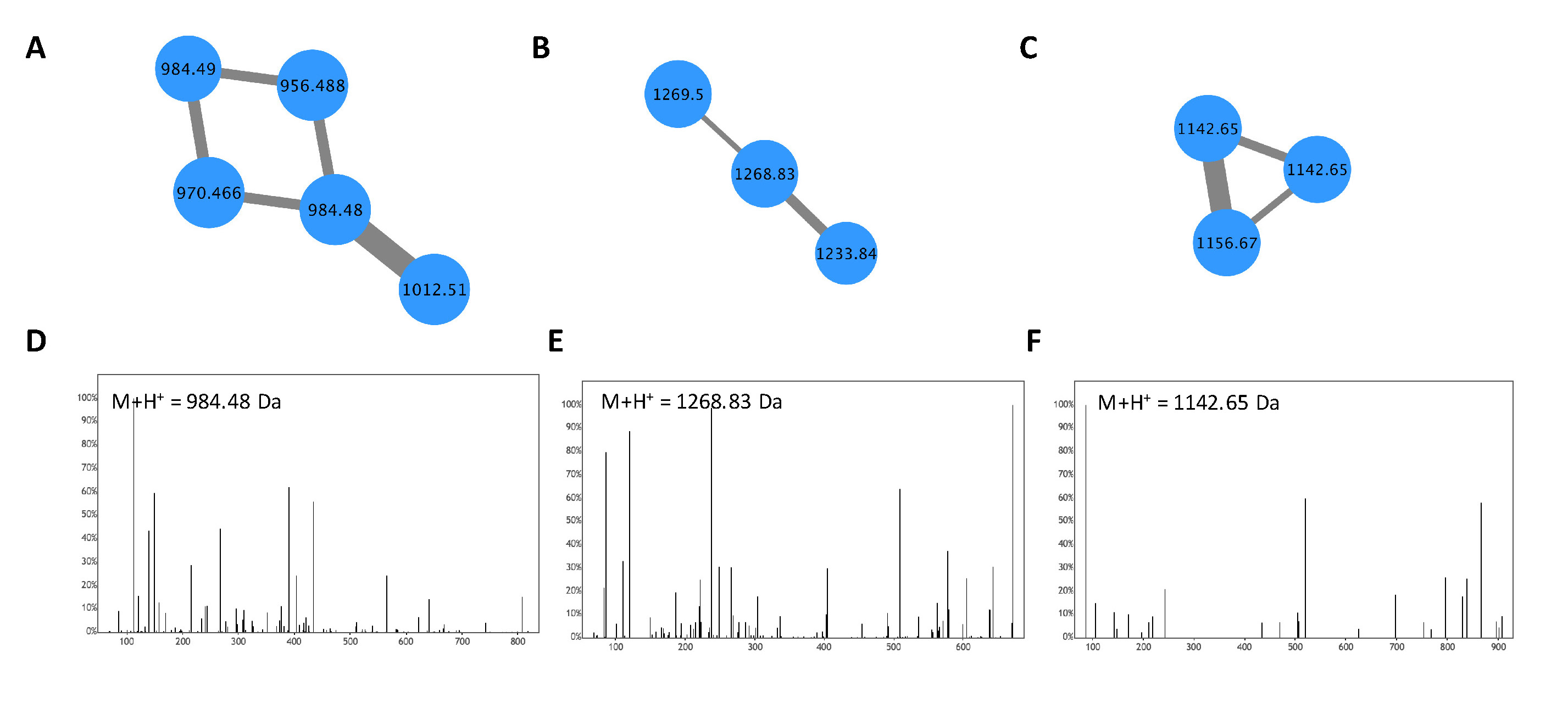

Supplement: FIGURE S5 — Unknown cluster “3–5” (A–C) highlighted by the GNPS analysis based on the MS/MS CID fragmentation spectra obtained from the 24 Microcystis strains. These clusters of uncharacterized molecules that present, respectively, intrinsic high fragmentation similarity (examples of representative spectra are provided D–F) may correspond to families of metabolites that still need to be characterized. Analytes detected in M. wessenbergii/viridis strains only are indicated in blue. [file Image_5.JPEG]
